# Evaluation of copper chloride crystallisation as a method for systems-level characterisation of phytopharmaceuticals – a pilot investigation

**DOI:** 10.1038/s41598-026-41081-6

**Published:** 2026-02-24

**Authors:** Greta Guglielmetti, Paul Doesburg, Claudia Scherr, David Martin, Stephan Baumgartner, Alexander L. Tournier

**Affiliations:** 1 Institute of Integrative Medicine, University of Witten/Herdecke, Herdecke, Germany; 2 Hiscia Research Institute, Society for Cancer Research, Arlesheim, Switzerland; 3 Institute of Complementary and Integrative Medicine, University of Bern, Bern, Switzerland

**Keywords:** Environmental sciences, Plant sciences

## Abstract

The multifaceted nature of phytotherapeutic products calls for methods able to provide a comprehensive and systems-level characterisation. Agricultural research suggests that copper chloride crystallisation (CCC) fingerprint analysis offers such possibilities. We therefore investigated the applicability of CCC to phytopharmaceutical questions. In this pilot trial, we analysed plant extracts of the same genus (*Viscum album* L.), featuring three progressively subtler differences: 1) subspecies (subsp. *album vs austriacum*), 2) deciduous host trees (apple *vs* oak), and 3) blending procedures (machine *vs* hand). In three sensitivity tests, we assessed CCC’s ability to detect these differences. CCC fingerprints were analysed using 7 variables characterising texture and structure. Systematic control experiments indicate that the setup is stable. In the *Verum* experiments, all variables passed the first sensitivity test (*p* < 0.01, Cohens’*d* 1.75–0.22). Four, mostly texture-related variables (*p* < 0.01, Cohens’*d* 0.56–0.26) and two structure variables (p < 0.01, Cohens’*d* 0.27, 0.20) passed the second and third sensitivity test, respectively. Our results demonstrated CCC’s ability, in our experimental setup, to detect differences between subspecies, deciduous host trees and blending procedures, although with progressively weaker statistical significance. Further development is needed to establish the relevance of CCC for phytopharmaceuticals and whether it can also detect systems-level properties.

**Supplementary Information:**

The online version contains supplementary material available at 10.1038/s41598-026-41081-6.

## Introduction

Phytotherapy has been utilised in many cultures throughout history, representing one of the oldest and most widespread forms of medicine^1–4^. Herbal products are widely used today, and their popularity is steadily growing^5–7^. This widespread usage underscores the importance of quality control to ensure the characterisation, efficacy and safety of phytotherapeutic products. Nevertheless, a debate is open about whether current assays, which primarily emphasise the quantitative measurement of single biomarkers, are always appropriate^8,9^. Although this strategy is essential for safety issues, and in some cases helpful for research on the mode of action, product characterisation might benefit from a complementary approach, providing a comprehensive and systems-level view of the products.

One reason is that phytotherapeutic products and medicinal plants possess complex chemical compositions and biological activities that often cannot be simplified into single-target models (i.e. one single substance interacting with a single target in the organism)^10–12^. Therefore, it could be interesting to aim at a broad overall characterisation of the products instead of focusing on single markers. This might be particularly helpful for products qualified as “other extracts” (Ph. Eur.^13^). Many of the products under this definition are well-established traditional herbal medicine products, but where no defined constituents with therapeutic activity are known, with the consequence that the active substance is the entire extract. In these cases, one can argue that it makes little sense to track only a few analytical markers whose therapeutic activity is not even supported by scientific evidence^8,9^. Once again, methods able to describe the products as a whole, aiming at characterising them in a comprehensive way, are desirable. Furthermore, the assessment of single or a few chemical compounds is a quite simplistic way to characterise all the underlying processes (e.g. environmental conditions of the medicinal plant and/or manufacturing procedures) which contribute to the final quality of the product.

Qualitative methodologies such as chromatographic/spectroscopic fingerprinting (e.g. HPTLC, ^1^HNMR) have already been introduced in the field with the aim of providing a comprehensive overview of the samples^9^. However, although these techniques allow for a broader range of investigation, they remain confined to the chemical level, offering a unilateral characterisation of the samples. To fully characterise samples, it would be advantageous to adopt methodologies with a broader scope that extend beyond the chemical profile, allowing for the exploration of systemic properties of the samples.

**Fig. 1:**
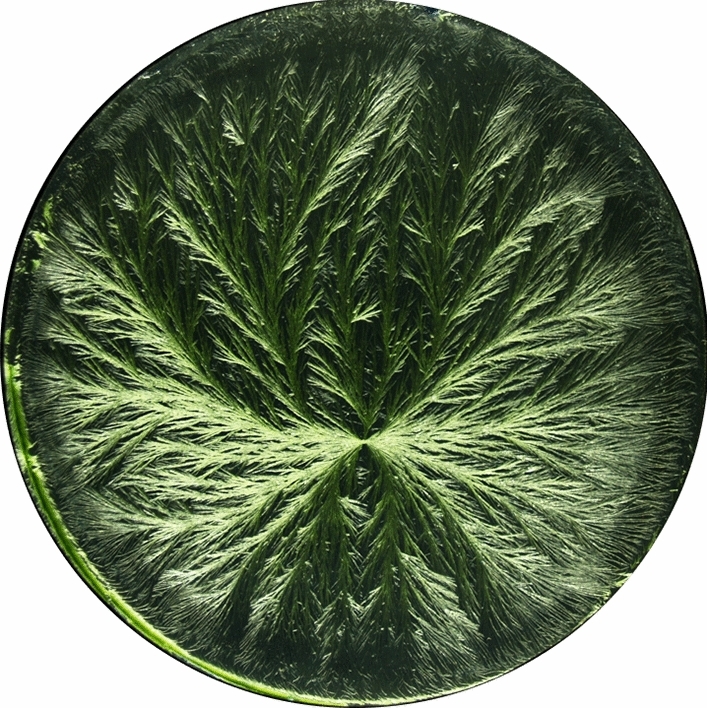
Photo of a representative fingerprint of the CCC method obtained from a watery solution of 150 mg of mistletoe extract and 150 mg CuCl_2_.

One method recognised for offering this characteristic is Copper Chloride Crystallisation (CCC)^14–16^. CCC is a methodology under the umbrella of metabolomic fingerprinting based on pattern formation^17^. The metabolome of the sample, defined as the whole array of chemical components (primary and secondary metabolites), including physical properties like surface tension and viscosity, is investigated in its entirety. The resulting two-dimensional, ramified crystallisation pattern morphology (hereafter referred to as a fingerprint) is analysed either via computer analysis or human visual evaluation (Panel trained according to DIN EN ISO 8586:2014) (see Fig. 1). This results in a characterisation of the fingerprints, either of structural and textural features in the case of computer analysis, or of qualitative descriptions (i.e. “gestures” and/or “gestalts”) in the case of human observers^18–21^.

The most interesting characteristic of the CCC method lies in its systems-level approach, which is reflected at different levels. Firstly, the sample is seen as a whole and therefore, it is examined in its entirety without selecting specific fractions, allowing for the full expression of the extract in the fingerprints. Secondly, the analysis of the fingerprints aims to provide a comprehensive descriptive overview of the multitude of features they display: from quantitative (e.g. length, width of crystallisation needles) to qualitative properties (e.g. integration, centre-coordination)^18–21^. Finally, the aspects that can be under the focus of investigation can range from those strictly related to the chemical composition (e.g. molecular weight, polysaccharides concentration) to systemic properties (e.g. resilience)^22–28^.

The ability of the CCC method to grasp systemic properties of the samples under investigation has been confirmed in the fields of food quality and agriculture, which have become this methodology’s two main areas of application. This stems from a growing interest in a systems-level approach to food quality, driven by the organic agriculture perspective and the need to better understand the nutrition–health relationship, e.g. via the food matrix^29^. Hence, a methodology that evaluates samples in their entirety, from the material composition to more nuanced factors such as freshness, resilience and ripeness, is required. Morphological and gestural characteristics of fingerprints obtained from progressively degraded food were found to mirror the ageing process of the samples. Using these degradation characteristics, fingerprints of agricultural samples derived from different farming systems were not only correctly grouped according to the farming system, but also ranked based on the perceived resilience of the samples^15,22,26,27,30,31^.

Considering the potential of CCC to characterise samples in their entirety and the encouraging results of CCC in agriculture and food quality, we set out to investigate the suitability and sensitivity of this method for the characterisation of phytotherapeutic products. The primary aim of this preliminary study was to assess the potential, if any, of CCC for applications in this field. In order to do so, we tested CCC on samples that allowed the investigation of three progressively finer differences.

**Fig. 2:**
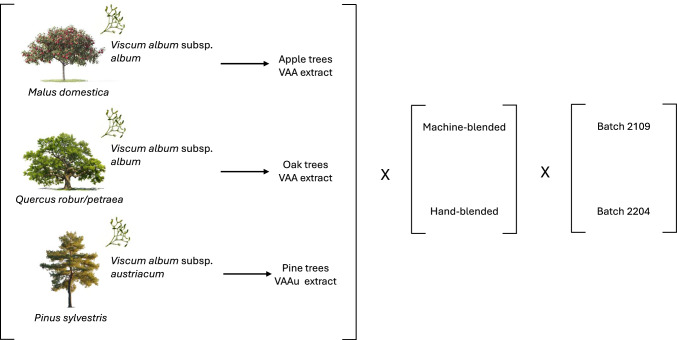
Schematic representation of the origin of the samples. The raw material is *Viscum album*. The host trees, biologically determined by the two subspecies selected for this study (i.e. subsp. *album* and subsp. *austriacum*), are apple, oak and pine trees. For each host tree, a corresponding mistletoe extract is obtained and processed by a specific blending machine or by hand. The samples were obtained from two subsequent productions (i.e. batch 2109, 2204).

We chose extracts from European mistletoe (*Viscum album* L.) as samples (Fig. 2). Mistletoe is a plant of great interest in phytotherapy and complementary medicine, particularly for its use in integrative cancer treatment^32–36^. It is a hemiparasitic shrub growing on different host trees. The mistletoe subspecies biologically determines the type of host tree on which it grows. For example, among some of the most common mistletoes used in therapy, *Viscum album* subsp. *album* (hereafter VAA) is bound to deciduous trees, and *Viscum album* subsp. *austriacum* (hereafter VAAu) is bound to *Pinus* sp.^37^. In terms of active components, there are significant chemical differences between different subspecies, whereas for mistletoes of the same subspecies but growing on different host trees, the difference is less pronounced^38,39^. This characteristic allowed us to perform the first two sensitivity tests: discrimination of mistletoe extracts 1) belonging to two different subspecies (i.e. VAA *vs* VAAu) and 2) belonging to the same subspecies (i.e. VAA) but growing on two different deciduous trees (i.e. apple *vs* oak trees). Finally, for the most challenging test, we aimed to determine whether CCC could identify a difference between two blending procedures applied to the extracts, a distinction that other metabolomic techniques had not been able to detect until now^40^. We compared blending by hand with blending using a specifically dedicated machine suggested by Rudolf Steiner, the founder of anthroposophic medicine^41^. According to anthroposophic pharmacy, the machine-based blending procedure aims at modifying systemic properties, thereby contributing to the effectiveness of the final product in cancer treatment^41–43^.

## Materials and methods

### General experimental procedure and design

Extracts of mistletoe of two different subspecies, grown on three different host trees and subjected to two blending procedures, were used as samples. The extracts were mixed with CuCl_2_ solution and pipetted on glass plates. Evaporation of the solution under controlled conditions led to the formation of CCC fingerprints. These were analysed by computer image analysis and subsequent statistical evaluation. The experimental design was structured to assess the sensitivity (i.e. ability to statistically discriminate between samples) of CCC by testing three progressively finer differences in the extracts: 1) mistletoe subspecies, 2) mistletoe belonging to the same subspecies but growing on different deciduous trees, and 3) blending procedures. As a first step, the stability of the setup and the randomisation of the samples in the CCC chambers were investigated with 6 systematic control (hereafter SC) experiments. Subsequently, 12 *Verum* experiments were run. Samples were block-randomised and blinded throughout the experiments, with decoding performed during the statistical analysis phase.

### Samples

The raw material was *Viscum album* L. subsp. *album* grown on apple trees (*Malus domestica* Borkh.) and oak trees (*Quercus robur* and *Quercus petraea*) as well as *V. album* L. subsp. *austriacum* (Wiesb.) Vollm. grown on pine trees (*Pinus sylvestris* L.). Mistletoe from each host tree yielded a distinct extract (Fig. 2).

The three extracts were prepared by blending the fermented juices obtained from the fresh material of winter and summer harvests of mistletoe grown on the corresponding host trees. Two different blending procedures were applied to the extracts. In one instance, the juices were combined by hand in a glass container, shaking it vertically 10 times to obtain a homogeneous extract called hand-blended. In the other instance, a machine specifically developed for this purpose at the Hiscia Institute (Society for Cancer Research, Arlesheim, Switzerland) was utilised. The result is the machine-blended extract (for more details, see^41^).

After the blending procedures, samples were sterile filtered, filled into 1 ml glass vials under sterile conditions and stored at 4 °C. 1 ml solution contains the fermented extract of 200 mg of fresh plant material. Two batches of two subsequent production lines in September 2021 and April 2022 were analysed (i.e. batch 2109 and 2204). Batch 2109 was made with 3-month-old summer juice from 2021 and 9-month-old winter juice from 2020/2021; batch 2204 was made with 3-month-old winter juice from 2021/2022 and 9-month-old summer juice from 2021. All samples were obtained from Iscador AG (Arlesheim, Switzerland).

The CCC experiments for this project were conducted between January and March 2023. A fresh stock solution for each sample was prepared daily by combining the contents of 20 vials. To summarise, 12 stock solutions per day were obtained: extracts derived by three different host trees (apple, oak and pine trees), obtained by two different mixing procedures (machine-blended, hand-blended) from two batches (2109, 2204).

No endangered or protected species were involved in this study. Experiments were conducted in accordance with institutional, national, and international guidelines and legislation.

### Copper chloride crystallisation (CCC)

Experiments were conducted in two crystallisation chambers, both located in the laboratory of the Hiscia Research Institute (Society for Cancer Research, Arlesheim, Switzerland). Both CCC chambers were constructed according to Andersen et al.^15,44^. Crystallisation solutions consisting of a mixture of a dihydrate copper chloride solution and the extracts were pipetted into Petri dishes. These were arranged in two concentric rings (43 allocations in total), within a crystallisation chamber with standardised temperature and humidity conditions (29 °C; initial rH 49%, air velocity 4 cm s^−1^; HygroClip S, Rotronic AG, Switzerland), allowed to evaporate and crystallise. The evaporation process of each allocation was monitored using time-lapse stills at 10-min intervals from a digital camera (Nikon D5000, objective AF-S DX Nikon 16-85 MB f/3.5–5.6G ED VR, Nikon, Tokyo, Japan) mounted on the ceiling of the crystallisation chamber. The crystallisation result is a thin-layer two-dimensional dendritic crystal pattern, grown of the bottom of the Petri dish. The glass plates that made up the Petri dishes were made of circular float glass (1st quality float glass; “air side” applied; 100 mm diameter; thickness 2.0 mm ± 0.2; Pfaehler GmbH & Co. KG, Gengenbach, Germany). The float glass was cleaned in a laboratory dishwasher in three washing steps: neodisher FLA, neodisher Z (both VWR, Radnor, USA), and deionised water at 90 °C. In order to build a basin in which to pipet the crystallisation solutions, the plates were combined with acrylic rings (made from GS acrylic tubes Riacryl; height 35 mm, thickness 5 mm ± 0.5; Schweigbauser Kunststoffe, Oberwil, Switzerland). Glass plates and acryl rings were glued with Vaseline (Sigma-Aldrich, St. Louis, MO, USA; CAS: 8009–03-8). On the day of the experiment, immediately after preparing the stock solution, the crystallisation solutions were also prepared, consisting of sample, CuCl_2_ 2H_2_O (copper (II) chloride dihydrate; PanReach Applichem, Darmstadt, Germany; article no. 131264.1211) and deionised water. Each sample was assigned four plates, i.e. repetitions here are technical replicates (see below for randomised sample plate allocation). In each plate, 6 mL crystallisation solution of the corresponding sample was pipetted, resulting in 150 mg sample and 150 mg CuCl_2_ per plate. For each plate, all the information about the sample and sample treatment was collected in a computer-based laboratory documentation system (LabDoc)^15^. For completeness, we mention the fact that in the *Verum* experiments, another sample concentration was also prepared for exploratory reasons. This was not considered in the analysis, as upon visual inspection, it showed suboptimal crystal expression.

### Computer analysis of the fingerprints

Crystallisation fingerprints were scanned in transmission mode (Umax PowerLook III scanner, Umax, Taiwan) after a minimum storage time of 24 h under controlled climatic conditions (26 °C, rH 44%). LCh conversion was obtained with an IT8.7 reference Dia (Silverfast IT8 Scanner Colour Calibration Target, Fuji transparency 35 mm, ISO 12641–1 compliant 1997). Two analyses were performed on the scanned fingerprints: texture and structure analysis^18,20^. As previously described by Busscher et al., dedicated software (ACIA) was used to perform the image analyses and merge the results with the sample information stored in the LabDoc database^15^. In our case, not only the results of the texture analysis but also those of the structure analysis were integrated in ACIA.

Texture analysis, as described by^45^, was applied to the fingerprints^18^. 15 s-order texture analysis variables were computed from a Grey Level Co-occurrence Matrix (one pixel offset in a horizontal and vertical direction, ROI (i.e. Region Of Interest) 0–100 of the plate. Based on previous literature^36,46^ and human relatability of the variables, 4 variables were selected:

- **cluster_shade**: measure of skewness (asymmetry) and uniformity of the fingerprints.
- **diagonal_moment**: measure of the roughness and smoothness of bright and dark areas of the fingerprints (positive values indicate that the bright areas of the fingerprints are rough, and dark areas are smooth).
- **entropy**: measure of the information content of the fingerprints measured as randomness of intensity distribution (a more complex fingerprint will have a higher value than a less complex one).
- **kappa**: measurement for the consistency of the fingerprints.

Furthermore, structure analysis, as developed by ^20^, was applied to the fingerprints. This analysis allowed the description of the morphological structure of the crystals at ROI 0–90%. In particular, the width and length of the needles characterising the crystals were measured, yielding 15 outcome measures each. Based on a correlation matrix selection, we excluded all those variables with a correlation coefficient higher than ± 0.7. Finally, the variables lend, l220, and l250 were selected as outcome variables.

- **lend**: measures the number of needle end-points.
- **l220**: measures the number of crystallisation needles with a length between 19.2 and 22 pixels.
- **l250**: measures the number of crystallisation needles with a length exceeding 22 pixels.

### Systematic control experiments, randomisation and coding

Systematic control (SC) experiments were performed to investigate the stability of the experimental setup. One extract – a machine-blended extract obtained from mistletoe grown on apple trees of a batch not included in the *Verum* experiments (batch 2209) – was crystallised in all allocations of both crystallisation chambers over 3 experimental days (6 experiments in total). Computerised image analysis was performed on the fingerprints, calculating the values of the seven variables described above for each fingerprint. The image analysis data of the three SC experiments were normalised to the day mean. A random number generator was used to create six allocation blocks (corresponding to the 6 samples per CCC chamber in the *Verum* experiment). Each block consisted of 4 allocations, i.e. 4 repetitions per sample. Randomisation schemes were pre-selected from a set of 2 million random permutations so as to minimise the standard deviation across the six group means. To rule out possible location effects in the *Verum* experiments, the final randomisation scheme was selected so as to yield no statistically significant differences across the groups (ANOVA test). Distinct randomisation schemes were created for each CCC chamber.

For the V*erum* experiments, the block randomisation was transferred from the SC experiments. Each CCC chamber was assigned one batch (i.e. batch 2109 or 2204). Samples were coded by a person not being involved in the experiments with letters from A to F for batch 2109 and from G to L for batch 2204. In the course of the 6 *Verum* experimental days, samples were rotated across the 6 randomisation blocks as complete counterbalancing strategy (i.e. per each experimental day, each sample was crystallised in a different randomisation block). Per experimental day, 48 fingerprints were obtained (24 fingerprints per CCC chamber), yielding 288 fingerprints over the six experimental days. The decoding was performed after finishing all the experiments but before the statistical evaluation.

### Data analysis

ANOVA was used to assess the influence of the different independent parameters. We performed three analyses of the data (for *Verum* and SC experiments) according to the three sensitivity tests. The grouping of the data and, therefore, the ANOVA independent parameters were selected according to the research questions of the three tests. Data of mistletoe extracts from the seven variables were analysed by means of 4-way ANOVA F-tests. Only main effects and first-level interactions (hereafter referred to as interactions) were taken into account. Pairwise comparisons were calculated with the LSD Fisher test only if the preceding F test was significant (p < 0.01). This procedure (protected Fisher’s LSD) gives a good safeguard against type I errors without being too conservative, i.e. it also gives good security against type II errors^47^. Complete data on the ANOVA analysis and LSD Fisher tests are provided in the supplementary information. The effect size was calculated as Cohens’*d* where 0.2 was considered a small effect, 0.5 a medium effect and 0.8 a large effect^48^. In order to avoid false positive results, we restricted the significance threshold to p < 0.01. The data were processed and statistically analysed using Python 3.10^49^ with the package scipy 1.15.2^50^ on a standard PC (Latitude 3440, Dell, Round Rock, USA).

## Results

### First sensitivity test: subspecies

Given that the chemical differences in active substances among mistletoe subspecies are detectable with standard analytical methods (i.e. HPLC)^51^, we addressed this as the easiest and first test to prove CCC sensitivity. To this aim, VAA samples were compared to VAAu samples. To this aim, we pooled the data obtained from the fingerprints of extracts of mistletoe growing on apple and oak trees and compared them with those obtained from the fingerprints of extracts of mistletoe growing on pine trees. All 288 fingerprints were used for this sensitivity test.

For the ANOVA statistical analysis of this question, we designated the following four factors as independent parameters: experimental day (1–6), batch (2109, 2204), process (machine-blended, hand-blended), and subspecies (VAA, VAAu).

**Fig. 3:**
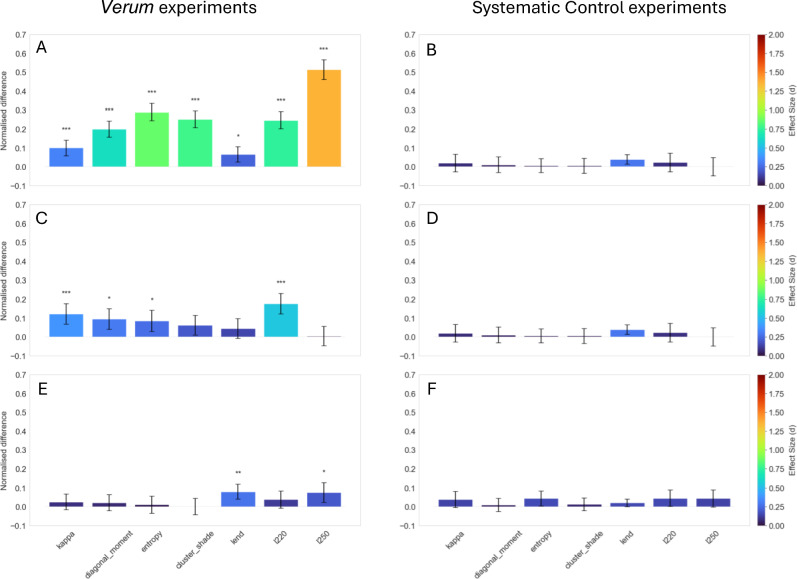
Normalised difference of ANOVA F-test main effects for the three sensitivity tests for *Verum* experiments (**A**,**C**,**E**) and corresponding systematic control experiments (**B**,**D**,**F**, respectively). Error bars represent 95% confidence intervals. Significant results are indicated as follows: * *p* < 0.01, ** *p* < 0.001, *** *p* < 0.0001. The colour of the bars indicates the effect size calculated as Cohen’s *d*.

The results of the SC experiments confirmed the stability of the experimental setup: no significant main effects were observed for the independent parameter in question (i.e. subspecies) in any of the seven variables (Fig. 3B). The only significant results were found for experimental day as main effect by kappa, entropy, lend and l250 (for complete data, see Supplementary).

Concerning the *Verum* experiments, as shown in Fig. 3A, a significant difference between the mistletoe subspecies was detected by all seven variables (p < 0.01). The main effect captured by diagonal_moment and l250 did not show any interaction, while all the other variables (i.e. kappa, cluster_shade, entropy, lend and l220) showed an interaction with the experimental day (Table 1). In particular, with regard to the variable entropy, although an interaction with the experimental day was evident, the significant difference between the subspecies was consistently observed. Whereas for cluster_shade, kappa and lend, a significant difference between the two subspecies was visible only during certain experimental days (day 1,2,4,5,6; day 1,5,6; day 2,4, respectively). Furthermore, entropy and kappa showed an additional interaction with the batch number. Despite the presence of a batch variation for entropy, a significant difference was observed between the two analysed subspecies. Kappa exhibited a significant difference between the two subspecies only in batch 2204. The effect size of the seven variables ranged between 1.76 for l250 and 0.23 for lend (Table 1).

**Table 1:** Summary of ANOVA F-test main effects and effect size (Cohen’s *d*) results for the three sensitivity tests. Significant results are indicated as follows: * *p* < 0.01, ** *p* < 0.001, *** *p* < 0.0001. Interactions are indicated as follows: d for experimental day, b for batch, p for blending procedure, h for host tree,—for no interaction. For complete data, see Supplementary.

Variable	First sensitivity test: subspecies			Second sensitivity test: deciduous trees			Third sensitivity test: blending procedures		
	*p*-value	Effect size	Interactions	*p*-value	Effect size	Interactions	*p*-value	Effect size	Interactions
kappa	< 0.0001***	0.33	d,b	< 0.0001***	0.38	b	0.1499	0.09	-
diagonal_moment	< 0.0001***	0.67	-	0.0011*	0.30	b	0.3460	0.07	b
entropy	< 0.0001***	0.95	d,b	0.0030*	0.26	b	0.6027	0.03	b
cluster_shade	< 0.0001***	0.84	d	0.0442	0.20	-	0.9645	0.00	b
lend	0.0056*	0.23	d	0.1015	0.14	p	0.0002**	0.28	h
l220	< 0.0001***	0.79	d	< 0.0001***	0.57	b	0.1061	0.12	-
l250	< 0.0001***	1.76	-	0.8803	0.02	d,b	0.0053*	0.20	-

### Second sensitivity test: deciduous trees

As a second sensitivity test, we explored the suitability of CCC in catching minor chemical differences^39^. In particular, we tested whether CCC was able to detect any difference in extracts of mistletoe belonging to the same subspecies but growing on different deciduous trees. We compared the data of the samples of mistletoe growing on apple vs oak trees, as both belong to the VAA subspecies. To this aim, the data of the samples of VAAu (i.e. mistletoe growing on pine trees) were excluded from the analysis. Only the 192 fingerprints obtained from samples of VAA subspecies were used for this sensitivity test, excluding the 96 fingerprints obtained from samples of VAAu subspecies.

For the ANOVA statistical analysis of this question, we designated the following four factors as independent parameters: experimental day (1–6), batch (2109, 2204), process (machine-blended, hand-blended), and deciduous tree (apple, oak).

The results of the SC experiments confirmed the stability of the experimental setup, as no significant main effect was observed for the independent parameter in question (i.e. deciduous tree) for any of the seven variables (Fig. 3D). Kappa, entropy, lend and l250 showed a significant main effect for the experimental day. Diagonal_moment and l220 did not show any significant result (for complete data, see Supplementary).

In Table 1, the results of the *Verum* experiments for this sensitivity test are summed up. Four out of seven variables showed a significant difference (p < 0.01) between mistletoe extracts grown on apple *vs* oak trees (Fig. 3C). All the variables (kappa, diagonal moment, entropy, l220) also showed significant interaction with the batch number. In particular, a significant difference between the two deciduous trees was visible for all four variables, only for batch 2204. The effect size range of the four variables (between 0.57 for l220 and 0.26 for entropy) in this sensitivity test was smaller compared to that observed in the first test.

### Third sensitivity test: blending procedures

The third and most subtle distinction was between samples prepared according to different pharmaceutical blending procedures. We selected a pharmaceutical blending procedure typical of anthroposophic pharmacy, which implies the use of a specific blending machine, and we compared it to blending by hand. For this test, we used the whole dataset. All 288 fingerprints were used for this sensitivity test.

For the ANOVA statistical analysis of this question, we designated the following four factors as independent parameters: experimental day (1–6), batch (2109, 2204), host tree (apple, oak and pine trees), and blending process (machine-blended, hand-blended).

The results of the SC experiments confirmed the stability of the experimental setup, as no significant main effect was observed for the independent parameter in question (i.e. pharmaceutical process) by any of the seven variables (Fig. 3F). The only notable results were kappa, entropy, lend and l250, showing a significant main effect for the experimental day. Diagonal_moment, l220 and cluster_shade did not show any significant result (for complete data, see Supplementary).

Table 1 shows the results obtained for this sensitivity test in the *Verum* experiments. Two out of seven variables showed a significant difference (l250 p = 0.0053, lend p = 0.0002 between the mistletoe extracts which underwent the machine-blended procedure vs the hand-blended procedure (Fig. 3E). In particular, the effect registered by l250 was stable throughout all the conditions (i.e. host tree, experimental day and batch), as it did not show any interaction. Whereas, the effect observed by lend shows a significant interaction with the host tree: the difference between the two pharmaceutical processes is visible only in samples of mistletoe grown on apple trees. Lastly, the effect size was 0.28 for lend and 0.20 for l250.

## Discussion

The need to find methods able to provide a comprehensive and systems-level characterisation of phytotherapeutic products led us to explore the use of CCC in the field of phytotherapy. We verified the suitability and sensitivity of the method by analysing extracts derived from plants of the same genus (i.e. *Viscum album* L.), featuring three progressively subtler differences: 1) mistletoe subspecies, 2) deciduous trees as mistletoe host trees, and 3) blending procedures. In order to assess these differences, three sensitivity tests were applied to the data obtained from the same set of experiments. The fingerprints were characterised using seven variables derived by traditional image analysis, describing their texture and structure. SC experiments were run to ensure the stability of the experimental setup.

The number of variables able to reach significance in each of the three sensitivity tests mirrored the increasing difficulty level of the tests. All seven variables demonstrated sensitivity to differences in the extracts in terms of mistletoe subspecies. Four variables were able to distinguish between extracts derived from mistletoe grown on apple *vs* oak trees, while only two variables indicated statistically significant differences attributable to the blending procedure.

The reliability of the results was confirmed by the SC experiments, which did not show any significant difference for any of the main effects in question (i.e. subspecies, deciduous trees, blending procedures). More specifically, kappa, entropy, lend and l250 exhibited a statistically significant effect only for the experimental day, while diagonal_moment, cluster_shade and l220 demonstrated stability across the three SC experiments, i.e. no main effects were observed even for the independent variables experimental day and batch. Moreover, it is important to note that the results of the SC experiments also ruled out possible differences between the CCC chambers. Indeed, none of the tests performed on the SC experiments showed a significant difference for the parameter batch, as main effect. Since in the SC experiments, only one sample was crystallised in both CCC chambers (i.e. no batch difference), this information has to be interpreted as indicating the equivalence of the two CCC chambers.

The first sensitivity test (i.e. subspecies difference), demonstrated an overall high statistical significance for all seven variables, consistent with the substantial difference in active constituents among mistletoe subspecies^38,52^. Noteworthy were diagonal_moment and l250, which showed no interactions with the subspecies differentiation. In terms of effect size, four out of the seven variables (i.e. entropy, cluster_shade, l220 and l250) performed well, showing a large effect size; one variable (i.e. diagonal_moment) showed a medium effect size, while only two variables (i.e. kappa and lend) showed a small effect size. The small effect sizes observed for kappa and lend can be attributed to statistically significant differences emerging on only a limited number of experimental days (three days for kappa and two days for lend). Additionally, in the case of kappa, subspecies differentiation was only possible for one batch (2204).

Concerning the second sensitivity level (i.e. deciduous tree difference), three of the four statistically significant variables were related to the texture of the fingerprints (i.e. kappa, diagonal_moment, entropy), and only one was related to the structure (i.e. l220). All four variables detected the difference between extracts of mistletoe grown on apple *vs* oak trees in only one batch (i.e. batch 2204). The overall lower effect sizes compared to those reached for the first sensitivity test mirror this batch interaction. Since the two batches were produced at different times of year, our findings might mirror seasonal variations in the chemical composition of the extracts.

Lastly, the third sensitivity test (i.e. blending procedure difference) was fulfilled only by variables related to the structure of the fingerprints (i.e. lend, l250). Interestingly, even though the investigated blending procedures are just subtly different, the variable l250 was able to detect the difference independently of all the other independent parameters. The variable lend could also detect the difference, but it showed a correlation with the host trees. More specifically, the blending procedure difference was visible only in the fingerprints of extracts obtained from mistletoe grown on apple trees. As expected, the effect sizes for this sensitivity test are smaller compared to those of the other two sensitivity tests.

In general, the fact that many statistically significant variables showed dependence on interactions, especially with batch and experimental days, indicates that the detection of such effects, while valid for our specific experimental setup, cannot yet be generalised.

To summarise, the best-performing variables according to statistical significance, effect size and interactions are the following. For the first sensitivity test, l250 (p < 0.0001, Cohen’s *d* 1.75, no interactions). For the second sensitivity test, considering that all the statistically significant variables detected a difference only in one batch, l220 showed the best performance (p < 0.0001, Cohen’s *d* 0.57). For the third sensitivity test, although lend showed a higher statistical power and effect size compared to l250, it showed interaction with experimental day, while l250 showed no interactions (lend: p < 0.0002, Cohen’s *d* 0.28; l250 p < 0.0053, Cohen’s *d* 0.20).

It should be noted that the above summary is provided only to facilitate a clearer understanding of the results. However, it would be inappropriate to limit this or future analyses to these variables. Relying on a limited set of variables for the analysis of the fingerprints carries the risk of missing potentially relevant signals. The second and third sensitivity tests show that capturing different aspects requires the use of different types of variables (i.e. predominantly texture-related variables vs structure-related variables, respectively for host trees and blending procedures discrimination). Most importantly, such a simplified approach would not align with the systems-level principle that CCC is based on, as it adopts the simplification mindset characteristic of single-target strategies. Applying CCC on phytotherapeutic products, we aim exactly to leverage this systems-level principle, which can offer “the holistic view that these complex products demand”^9^.

Based on the results obtained with our experimental setup, we identified two key advantages of CCC. Firstly, out of the same fingerprints, we could extract information about three different characteristics of the samples (i.e. subspecies, deciduous trees, blending procedures). Usual analytical tests used for characterisation (e.g. chromatography) are designed to analyse isolated constituents (i.e. specific biomarkers). For this reason, several tests of this kind are needed to ensure a reliable characterisation of the product^8,53,54^. In contrast, in our experiments, CCC fingerprints have shown the ability to mirror simultaneously three aspects of the samples, suggesting efficiency and versatility of the method with our experimental conditions. Secondly, CCC showed a quite interesting degree of sensitivity as it could, albeit weakly and with interactions, fulfil the third sensitivity test. A difference between the investigated blending procedures has already been investigated: in vitro bioassays showed an improved efficacy of the machine-blended extracts with respect to hand-blended extracts, but the nature of the difference remains unclear^41^. A recent study using metabolomics could not find any difference between the two processes in the investigated chemical spectrum^40^. The fact that CCC could detect a difference suggests there may be other options. One possibility is that the difference is on a chemical level, but in a chemical range different from the one previously investigated. Another possibility, considering the technical characteristics of the machine used for the blending procedure (e.g. high rotational speed), might be that the difference is present on a physical (structural) level. Lastly, it is possible that CCC captured a property of the samples that is not strictly confined either to the chemical or physical level but instead lies at the systems level. This might be a plausible option as the potential of CCC to grasp such systemic properties was assessed by food quality and agricultural studies (i.e. resilience to ageing). Moreover, the in vitro study that identified an efficacy difference between the two blending procedures assessed it in terms of resilience to intoxication^41^. Nevertheless, the hypothesis that CCC could have caught a system-level property, given the current state of the research, remains speculative.

Concerning the limitations of the current experimental design, the evaluation of the fingerprints was confined to computer analysis, missing the richer qualitative description obtainable through human observation. This can be overcome in future by means of a human visual evaluation panel (trained according to DIN EN ISO 8586:2014^19^). A second aspect regards the SC experiments. We performed these experiments with the subsequent allocation of spatial positions to fixed blocks and with a counterbalancing design to aim for a setup free of space-related systematic errors. However, in future experiments, the stability of the experimental design should be assessed with full sets of SC experiments after having set up the spatial experimental arrangements (i.e. equal number of SC and *Verum* experiments). As a third aspect, further investigations are needed to clarify the day and batch interactions observed, especially regarding the results of the second sensitivity test. In particular, concerning the batch, one possibility would be to repeat the experiments with different batches across several harvests/years to check batch variability. Nevertheless, information is also missing regarding the variability of the method with such samples; therefore, this issue should be solved first. For example, comparing different batches made with the same raw material.

Since this study is – to the best of our knowledge – the first one on the applicability of CCC for phytopharmaceutical questions, there are several possible future perspectives. We would deem it interesting to explore the general applicability of CCC on a broader range of phytotherapeutic products with a view to its possible future use in quality control (e.g. for identification, quality, stability etc.). In this context, the next essential step is the analytical validation of the method, which would also enable us to identify the sample parameters encoded by our variables. It would be also interesting to test extracts from different plants and compare them over several batches to assess the specificity and reproducibility of the CCC method. Another question would be how different extraction methods (e.g. infusions, macerations, percolations) and/or processing techniques appear on CCC fingerprints across different medicinal plants. Other interesting perspectives might be in the exploration of the further potential of CCC regarding “quality”. In agricultural research, CCC has been applied to test the resilience of samples against stress (e.g. ageing), which can be seen as a certain aspect of product quality. Correspondingly, for phytopharmaceutical research, the next necessary step would be finding and applying appropriate stress factors and testing phytopharmaceutical samples before and after stress application. This might provide information regarding the ability of CCC to catch resilience in phytotherapeutic products with regard to stress, which could be seen as a complementary aspect of quality of phytopharmaceutical extracts. Lastly, it would be worth focusing on the image analysis of the fingerprints to explore the full range of descriptive ability of the method. At the moment, the standard analyses are computer-based traditional image analysis and human visual evaluation. The introduction of machine learning with both supervised and unsupervised approaches might bridge the two, potentially enriching the descriptive palette together with more sophisticated analysis, such as multivariate analysis using supervised classification. Machine learning allows for more comprehensive analysis than traditional computer techniques, and it might catch morphological details that human vision might not notice ^55^.

## Conclusion

This article explores the potential applicability of CCC as a complementary method for characterising phytotherapeutic products, recognising their complex and multifaceted nature. As this methodology had not previously been applied in this field, our primary aim was to assess its sensitivity by testing its ability to detect differences between closely related phytotherapeutic samples.

We set up three sensitivity tests aiming to detect progressively finer differences between samples derived from plants of the same genus (i.e. *Viscum album*). Statistical differences were observed between the samples across the three tests, suggesting the differentiation ability of the method. Notably, the signals related to the three sensitivity tests were all extracted from the same set of experiments, hinting at the potential ability of the fingerprints to reflect multiple aspects of the products.

Further studies aim to extend this preliminary work by conducting analytical validation together with a more comprehensive analysis of the fingerprints. In this way, we aim to validate the pharmaceutical relevance of CCC, particularly its potential for system-level characterisation.

## Supplementary Information

Supplementary Information.

## Data Availability

The data related to the seven calculated variables are available on Zenodo https://zenodo.org/records/16919252.
