# Supplementary material for: Evaluation of copper chloride crystallisation as a method for systems-level characterisation of phytopharmaceuticals – a pilot investigation
Source: Sci Rep. 2026 Feb 24;16:7506. doi: 10.1038/s41598-026-41081-6 (PMC12932814; doi:10.1038/s41598-026-41081-6)
Supplement: Supplementary file 1 — Supplementary Information. [file 41598_2026_41081_MOESM1_ESM.pdf]

	Verum – First sensitivity test: Subspecies													
	**kappa**		**diagonal_moment**		**entropy**		**cluster_shade**		**lend**		**l220**		**l250**	
	**F**	***p***	**F**	***p***	**F**	***p***	**F**	***p***	**F**	***p***	**F**	***p***	**F**	***p***
**intercept**	45861	< 0.0001	982	< 0.0001	1.05E+07	< 0.0001	1928	< 0.0001	34519	< 0.0001	36586	< 0.0001	23250	< 0.0001
**subspecies**	20.8	< 0.0001	58.3	< 0.0001	126	< 0.0001	86.8	< 0.0001	7.82	0.006	78.02	< 0.0001	288	< 0.0001
**procedure**	0.213	0.645	0.196	0.659	0.988	0.321	0.166	0.684	7.83	0.006	2.87	0.092	3.11	0.079
**batch**	315	< 0.0001	224	< 0.0001	131	< 0.0001	179	< 0.0001	173	< 0.0001	132	< 0.0001	42.4	< 0.0001
**exp. day**	20.2	< 0.0001	1.85	0.104	19.8	< 0.0001	3.81	0.002	21.3	< 0.0001	12.4	< 0.0001	6.61	< 0.0001
**subspecies : procedure**	4.90	0.028	1.33	0.250	2.62	0.107	1.81	0.180	3.09	0.080	0.90	0.344	4.91	0.028
**subspecies : batch**	10.1	0.002	3.11	0.079	8.46	0.004	0.459	0.499	1.65	0.201	6.11	0.014	0.144	0.704
**procedure : batch**	1.53	0.218	4.11	0.044	9.37	0.002	6.19	0.013	1.04	0.310	3.23E-04	0.986	3.00	0.085
**subspecies : exp. day**	3.71	0.003	2.49	0.032	3.18	0.009	3.39	0.006	4.62	4.84E-04	3.52	0.004	1.13	0.347
**procedure : exp. day**	0.695	0.629	0.754	0.584	1.29	0.269	0.680	0.639	0.902	0.480	1.57	0.170	1.08	0.370
**batch : exp. day**	2.60	0.026	1.81	0.111	3.87	0.002	1.62	0.157	2.57	0.027	1.89	0.097	6.02	< 0.0001
**subspecies : procedure : batch**	1.79	0.182	1.56	0.213	0.668	0.414	1.03	0.310	0.001	0.973	0.55	0.461	0.973	0.325
**subspecies : procedure : exp. day**	0.962	0.442	1.05	0.388	1.58	0.167	1.73	0.128	0.224	0.952	0.97	0.436	0.167	0.975
**subspecies : batch : exp. day**	3.53	0.004	2.19	0.056	2.62	0.025	2.71	0.021	1.44	0.212	1.32	0.254	1.40	0.224
**procedure : batch : exp. day**	1.10	0.361	0.506	0.772	0.148	0.980	0.219	0.954	0.703	0.622	1.43	0.213	2.19	0.056
**subspecies : procedure : batch : exp. day**	0.83	0.526	1.02	0.408	0.284	0.922	0.979	0.431	0.581	0.715	1.07	0.375	0.62	0.687

Table S1: Results of the *Verum* First sensitivity test from ANOVA F-test (main effects and interactions). Independent experimental parameters were experimental day (1–6), batch (2109, 2204), procedure (machine-blended, hand-blended), and subspecies (VAA, VAAu).

		**kappa**	**entropy**	**cluster_shade**	**lend**	**l220**
**subspecies : batch**	**batch 2109**	0.233	< 0.0001	-	-	-
	**batch 2204**	< 0.0001	< 0.0001	-	-	-
**subspecies : exp. day**	**day 1**	< 0.0001	< 0.0001	< 0.0001	0.438	< 0.0001
	**day 2**	0.812	< 0.0001	< 0.0001	< 0.0001	0.003
	**day 3**	0.783	0.001	0.203	0.273	< 0.0001
	**day 4**	0.378	0.004	0.003	2.45E-04	0.187
	**day 5**	< 0.0001	< 0.0001	< 0.0001	0.019	< 0.0001
	**day 6**	0.008	< 0.0001	< 0.0001	0.889	1.05E-04

Table S2: *p*-values (Fisher-LSD test) of first-level interactions for the statistically significant main effects obtained in Table S1 (only significant interactions reported).

	Systematic Control – First sensitivity test: Subspecies													
	**kappa**		**diagonal_moment**		**entropy**		**cluster_shade**		**lend**		**l220**		**l250**	
	**F**	***p***	**F**	***p***	**F**	***p***	**F**	***p***	**F**	***p***	**F**	***p***	**F**	***p***
**intercept**	29425	< 0.0001	360	< 0.0001	6158463	< 0.0001	80.4	< 0.0001	31992	< 0.0001	12704	< 0.0001	16355	< 0.0001
**subspecies**	0.285	0.594	0.094	0.759	3.80E-04	0.984	0.126	0.722	1.48	0.225	0.113	0.737	0.002	0.967
**procedure**	1.03	0.312	0.017	0.897	2.61	0.108	0.030	0.861	2.05	0.153	1.700	0.194	2.84	0.093
**batch**	1.02	0.314	3.871	0.050	0.703	0.402	4.91	0.028	3.37	0.068	4.50	0.035	0.355	0.552
**exp. day**	27.4	< 0.0001	1.55	0.215	28.7	< 0.0001	2.25	0.108	9.05	1.65E-04	0.011	0.989	27.0	< 0.0001
**subspecies : procedure**	0.053	0.817	0.343	0.559	0.691	0.407	0.580	0.447	0.534	0.466	0.248	0.619	2.30	0.131
**subspecies : batch**	0.744	0.389	2.20	0.140	0.776	0.379	1.73	0.190	0.016	0.900	0.006	0.936	0.377	0.540
**procedure : batch**	1.04	0.309	2.16	0.143	0.836	0.361	2.77	0.097	1.11	0.293	3.16	0.077	4.18	0.042
**subspecies : exp. day**	1.72	0.181	0.090	0.914	1.20	0.302	0.275	0.760	0.175	0.840	1.30	0.276	0.055	0.946
**procedure : exp. day**	0.192	0.825	0.038	0.963	0.376	0.687	0.034	0.967	1.53	0.218	0.325	0.723	1.82	0.165
**batch : exp. day**	0.666	0.515	3.64	0.028	2.10	0.125	3.543	0.031	0.436	0.647	1.51	0.224	3.42	0.035
**subspecies : procedure : batch**	3.56	0.060	1.27	0.261	1.88	0.171	1.638	0.202	0.924	0.338	1.00	0.318	1.25	0.265
**subspecies : procedure : exp. day**	0.629	0.534	0.988	0.374	0.365	0.695	0.531	0.589	0.779	0.460	0.758	0.470	1.21	0.301
**subspecies : batch : exp. day**	0.481	0.619	1.68	0.188	1.23	0.295	1.193	0.305	0.627	0.535	0.128	0.880	0.489	0.614
**procedure : batch : exp. day**	0.718	0.489	0.141	0.869	0.764	0.467	0.067	0.935	1.59	0.206	1.75	0.175	1.51	0.223
**subspecies : procedure : batch : exp. day**	0.578	0.562	0.441	0.644	1.09	0.338	0.536	0.586	3.80	0.024	3.09	0.047	2.26	0.107

Table S3: Results of the Systematic Control First sensitivity test from ANOVA F-test (main effects and interactions). Independent experimental parameters were experimental day (1–6), batch (2109, 2204), procedure (machine-blended, hand-blended), and subspecies (VAA, VAAu).

	Verum – Second sensitivity test: Deciduous trees													
	**kappa**		**diagonal_moment**		**entropy**		**cluster_shade**		**lend**		**l220**		**l250**	
	**F**	***p***	**F**	***p***	**F**	***p***	**F**	***p***	**F**	***p***	**F**	***p***	**F**	***p***
**intercept**	51066	< 0.0001	474	< 0.0001	8606909	< 0.0001	941	< 0.0001	27095	< 0.0001	37375	< 0.0001	14696	< 0.0001
**deciduous tree**	33.1	< 0.0001	11.2	0.001	9.12	0.003	4.12	0.044	2.72	0.101	44.7	< 0.0001	0.023	0.880
**procedure**	7.63	0.006	2.15	0.145	0.322	0.571	0.69	0.408	15.8	1.10E-04	0.622	0.432	12.7	5.00E-04
**batch**	467	< 0.0001	236	< 0.0001	169	< 0.0001	155	< 0.0001	158	< 0.0001	218	< 0.0001	38.0	< 0.0001
**exp. day**	33.5	< 0.0001	5.39	1.43E-04	23.3	< 0.0001	8.70	< 0.0001	28.1	< 0.0001	21.9	< 0.0001	8.34	< 0.0001
**deciduous tree : procedure**	2.48	0.117	5.58	0.020	0.891	0.347	3.43	0.066	22.8	< 0.0001	0.024	0.878	2.61	0.108
**deciduous tree : batch**	34.5	< 0.0001	8.27	0.005	23.9	< 0.0001	5.35	0.022	1.05	0.307	27.1	< 0.0001	17.6	< 0.0001
**procedure : batch**	7.07	0.009	9.05	0.003	12.4	5.83E-04	9.67	0.002	0.737	0.392	0.644	0.424	5.91	0.016
**deciduous tree : exp. day**	1.67	0.146	0.830	0.530	1.81	0.115	0.825	0.534	1.16	0.331	1.91	0.095	3.29	0.008
**procedure : exp. day**	1.33	0.255	1.49	0.197	1.95	0.090	2.36	0.043	0.981	0.432	0.622	0.683	1.28	0.278
**batch : exp. day**	4.40	9.33E-04	1.36	0.243	4.85	3.98E-04	1.78	0.120	1.89	0.099	3.57	0.005	5.70	< 0.0001
**deciduous tree : procedure : batch**	0.008	0.928	7.04	0.009	2.74	0.100	4.87	0.029	1.93	0.167	1.01	0.317	0.985	0.323
**deciduous tree : procedure : exp. day**	2.14	0.064	1.32	0.259	0.581	0.715	0.730	0.602	0.727	0.604	0.801	0.550	2.21	0.056
**deciduous tree : batch : exp. day**	0.348	0.883	0.860	0.510	0.401	0.848	0.767	0.576	1.12	0.351	0.540	0.746	0.113	0.989
**procedure : batch : exp. day**	0.627	0.679	0.327	0.896	0.312	0.905	0.608	0.694	1.19	0.316	1.40	0.228	0.746	0.590
**deciduous tree: procedure : batch : exp. day**	2.526	0.032	2.62	0.026	2.06	0.073	2.06	0.073	0.895	0.486	1.66	0.149	0.487	0.786

Table S4: Results of the *Verum* Second sensitivity test from ANOVA F-test (main effects and interactions). Independent experimental parameters were experimental day (1–6), batch (2109, 2204), procedure (machine-blended, hand-blended), and deciduous tree (apple trees, oak trees).

		**kappa**	**diagonal_moment**	**entropy**	**l220**
**deciduous tree: batch**	**batch 2109**	0.934	0.741	0.189	0.298
	**batch 2204**	< 0.0001	< 0.0001	< 0.0001	< 0.0001

Table S5: *p*-values (Fisher-LSD test) of first-level interactions for the statistically significant main effects obtained in Table S4 (only significant interactions reported).

	Systematic Control – Second sensitivity test: Deciduous trees													
	**kappa**		**diagonal_moment**		**entropy**		**cluster_shade**		**lend**		**l220**		**l250**	
	**F**	***p***	**F**	***p***	**F**	***p***	**F**	***p***	**F**	***p***	**F**	***p***	**F**	***p***
**intercept**	26910	< 0.0001	257	< 0.0001	8630063	< 0.0001	53.0	< 0.0001	24760	< 0.0001	10565	< 0.0001	17332	< 0.0001
**deciduous tree**	0.267	0.606	0.074	0.787	0.063	0.803	0.022	0.883	3.81	0.053	0.271	0.604	1.73E-04	0.990
**procedure**	1.41	0.238	0.376	0.541	0.859	0.356	0.627	0.430	0.376	0.541	0.538	0.465	0.030	0.863
**batch**	3.18	0.077	0.173	0.678	4.14	0.044	0.579	0.448	2.23	0.137	3.44	0.066	3.53E-04	0.985
**exp. day**	14.2	< 0.0001	1.09	0.338	25.6	< 0.0001	2.33	0.101	8.92	2.23E-04	0.897	0.410	26.3	< 0.0001
**deciduous tree : procedure**	0.030	0.863	0.295	0.588	0.410	0.523	0.741	0.391	0.156	0.694	0.870	0.353	0.075	0.784
**deciduous tree : batch**	1.91	0.169	0.025	0.874	0.425	0.516	0.005	0.944	0.174	0.677	0.192	0.662	0.330	0.567
**procedure : batch**	0.685	0.409	0.087	0.768	0.293	0.589	0.106	0.746	0.006	0.935	0.496	0.482	0.907	0.342
**deciduous tree : exp. day**	1.10	0.335	0.552	0.577	0.967	0.383	0.382	0.683	0.840	0.434	0.069	0.933	1.45	0.238
**procedure : exp. day**	1.23	0.294	0.533	0.588	0.395	0.675	0.536	0.587	0.156	0.856	0.667	0.515	0.359	0.699
**batch : exp. day**	0.029	0.972	0.276	0.759	0.891	0.412	0.645	0.526	1.34	0.264	0.793	0.454	5.65	0.004
**deciduous tree : procedure : batch**	0.247	0.620	0.059	0.808	0.946	0.332	0.006	0.940	0.562	0.455	0.060	0.807	0.135	0.713
**deciduous tree : procedure : exp. day**	0.176	0.838	0.067	0.936	0.249	0.780	0.047	0.954	0.787	0.457	0.122	0.885	0.397	0.673
**deciduous tree : batch : exp. day**	0.744	0.477	0.602	0.549	0.577	0.563	0.730	0.484	0.896	0.411	0.090	0.914	0.127	0.881
**procedure : batch : exp. day**	0.059	0.943	0.476	0.622	0.739	0.480	0.574	0.564	2.21	0.113	0.250	0.779	1.52	0.222
**deciduous tree: procedure : batch : exp. day**	0.121	0.886	0.057	0.945	2.27	0.107	0.186	0.830	2.05	0.133	0.571	0.566	1.62	0.202

Table S6: Results of the Systematic Control Second sensitivity test from ANOVA F-test (main effects and interactions). Independent experimental parameters were experimental day (1–6), batch (2109, 2204), procedure (machine-blended, hand-blended) and deciduous tree (apple trees, oak trees).

	***Verum* – Third sensitivity test: Blending procedures**													
	**kappa**		**diagonal_moment**		**entropy**		**cluster_shade**		**lend**		**l220**		**l250**	
	**F**	***p***	**F**	***p***	**F**	***p***	**F**	***p***	**F**	***p***	**F**	***p***	**F**	***p***
**intercept**	67580	< 0.0001	1081	< 0.0001	1.38E+07	< 0.0001	2001	< 0.0001	43959	< 0.0001	49272	< 0.0001	27352	< 0.0001
**procedure**	2.09	0.150	0.892	0.346	0.272	0.603	0.002	0.965	14.4	1.89E-04	2.63	0.106	7.95	0.005
**host tree**	28.5	< 0.0001	39.5	< 0.0001	78.8	< 0.0001	48.5	< 0.0001	5.88	0.003	66.6	< 0.0001	163	< 0.0001
**batch**	514	< 0.0001	315	< 0.0001	203	< 0.0001	222	< 0.0001	233	< 0.0001	210	< 0.0001	55.9	< 0.0001
**exp. day**	33.6	< 0.0001	3.66	0.003	28.9	< 0.0001	6.57	< 0.0001	32.2	< 0.0001	19.4	< 0.0001	9.68	< 0.0001
**procedure : host tree**	4.29	0.015	3.64	0.028	2.01	0.14	2.71	0.069	14.3	< 0.0001	0.565	0.569	4.15	0.017
**procedure : batch**	4.11	0.044	7.78	0.006	14.6	1.70E-04	9.61	0.002	1.28	0.260	0.097	0.756	5.38	0.021
**host tree : batch**	22.2	< 0.0001	6.06	0.003	17.7	< 0.0001	2.97	0.053	1.50	0.225	15.0	< 0.0001	9.40	1.22E-04
**procedure : exp. day**	0.965	0.44	1.12	0.352	1.71	0.133	1.28	0.272	1.24	0.293	1.41	0.222	1.54	0.177
**host tree : exp. day**	3.15	8.72E-04	1.87	0.050	2.82	0.003	2.23	0.017	3.23	6.75E-04	2.96	0.002	2.37	0.011
**batch : exp. day**	3.37	0.006	1.64	0.150	5.22	1.52E-04	1.48	0.198	2.80	0.018	2.81	0.018	7.71	< 0.0001
**procedure : host tree : batch**	1.16	0.315	4.53	0.012	1.85	0.159	3.03	0.050	1.07	0.356	0.754	0.472	1.07	0.345
**procedure : host tree : exp. day**	1.59	0.11	1.29	0.238	1.23	0.271	1.30	0.233	0.53	0.870	0.929	0.507	1.26	0.253
**procedure : batch : exp. day**	1.08	0.37	0.31	0.905	0.21	0.956	0.22	0.952	1.09	0.369	1.571	0.169	2.07	0.070
**host tree : batch : exp. day**	2.44	0.009	1.71	0.080	1.75	0.071	1.84	0.056	1.42	0.171	1.040	0.411	0.850	0.581
**procedure : host tree : batch : exp. day**	1.68	0.086	1.94	0.041	1.27	0.25	1.57	0.116	0.82	0.610	1.346	0.207	0.606	0.808

Table S7: Results of the *Verum* Third sensitivity test from ANOVA F-test (main effects and interactions). Independent experimental parameters were experimental day (1–6), batch (2109, 2204), host tree (apple trees, oak trees, pine trees) and procedure (machine-blended, hand-blended).

		**lend**
**procedure : host tree**	**Apple trees**	< 0.0001
	**Oak trees**	0.555
	**Pine trees**	0.502

Table S8: *p*-values (Fisher-LSD test) of first-level interactions for the statistically significant main effects obtained in Table S7 (only significant interactions reported).

	Systematic control – Third sensitivity test: Blending procedures													
	**kappa**		**diagonal_moment**		**entropy**		**cluster_shade**		**lend**		**l220**		**l250**	
	**F**	***p***	**F**	***p***	**F**	***p***	**F**	***p***	**F**	***p***	**F**	***p***	**F**	***p***
**intercept**	32211	< 0.0001	385	< 0.0001	6720879	< 0.0001	84.8	< 0.0001	36425	< 0.0001	13735	< 0.0001	17863	< 0.0001
**procedure**	1.30	0.256	0.114	0.736	1.95	0.164	0.200	0.655	1.60	0.207	1.40	0.238	1.52	0.219
**host tree**	0.255	0.783	0.081	0.922	0.016	0.984	0.072	0.931	2.63	0.074	0.172	0.842	8.77E-04	0.999
**batch**	1.83	0.177	2.35	0.127	1.40	0.238	3.42	0.066	3.65	0.057	4.73	0.031	0.167	0.683
**exp. day**	25.2	< 0.0001	1.63	0.199	27.2	< 0.0001	2.73	0.067	11.2	< 0.0001	0.087	0.916	28.7	< 0.0001
**procedure : host tree**	0.038	0.963	0.309	0.735	0.442	0.643	0.654	0.521	0.346	0.708	0.497	0.609	1.14	0.321
**procedure : batch**	0.167	0.683	1.30	0.256	0.228	0.633	1.66	0.199	0.611	0.435	2.25	0.135	3.05	0.082
**host tree : batch**	1.13	0.326	1.07	0.356	0.487	0.615	0.836	0.435	0.094	0.910	0.087	0.917	0.296	0.744
**procedure : exp. day**	0.481	0.619	0.051	0.950	0.287	0.751	0.167	0.846	1.04	0.355	0.342	0.711	1.15	0.317
**host tree : exp. day**	1.28	0.280	0.313	0.869	0.835	0.504	0.325	0.861	0.504	0.733	0.652	0.629	0.525	0.718
**batch : exp. day**	0.380	0.684	2.35	0.098	1.458	0.235	2.597	0.077	0.836	0.435	1.39	0.249	4.31	0.015
**procedure : host tree : batch**	1.83	0.163	0.637	0.530	1.159	0.316	0.791	0.454	0.743	0.477	0.507	0.603	0.654	0.521
**procedure : host tree : day_date**	0.38	0.826	0.507	0.731	0.242	0.914	0.210	0.891	0.782	0.538	0.417	0.797	0.722	0.578
**procedure : batch : day_date**	0.406	0.667	0.228	0.796	0.483	0.617	0.21	0.811	1.34	0.264	0.629	0.534	1.08	0.343
**host_tree : batch : day_date**	0.532	0.713	1.101	0.357	0.746	0.562	0.943	0.440	0.759	0.553	0.100	0.982	0.281	0.890
**procedure : host tree : batch : day_date**	0.329	0.858	0.240	0.916	1.12	0.349	0.352	0.842	2.930	0.022	1.73	0.144	1.65	0.163

Table S9: Results of the Systematic Control Third sensitivity test from ANOVA F-test (main effects and interactions). Independent experimental parameters were experimental day (1–6), batch (2109, 2204), host tree (apple trees, oak trees, pine trees) and procedure (machine-blended, hand-blended).
